# Trend and socio-demographic differentials of Caesarean section rate in Addis Ababa, Ethiopia: analysis based on Ethiopia demographic and health surveys data

**DOI:** 10.1186/1742-4755-11-14

**Published:** 2014-02-14

**Authors:** Samson Gebremedhin

**Affiliations:** 1 School of Public and Environmental Health, Hawassa University, Hawassa, Ethiopia

**Keywords:** Caesarean section, Maternal health, Addis Ababa

## Abstract

**Background:**

According to the World Health Organization, Caesarean Section (CS) rate (percentage of births managed by CS) exceeding 15% lacks medical justification and it could be linked with adverse maternal and child health consequences. Nonetheless, the rate in Addis Ababa city is beyond the aforementioned level. The objectives of the study were to assess the trend and socio-demographic differentials of CS rate in the city.

**Methods:**

The study was made based on the three Ethiopia Demographic and Health Surveys (EDHS) data (EDHS 2000, 2005 and 2011). The trend over the period of 1995–2010 was assessed using simple linear regression analysis whereas the differentials of CS rate were identified based on DHS 2011 data. CS rates were compared across categories of various socio-economic variables using chi-square test.

**Results:**

The CS rate increased significantly from 2.3% in 1995–1996 to 24.4% in 2009–2010. From 2003 onwards, it persisted above 15%. The rates among women with secondary (32.3%) or higher (33.3%) levels of education were nearly two times higher than the corresponding figures in the illiterates (14.8%) and women with primary education (15.8%) (*P < 0.001*). The level among women from the ‘rich’ households (28.6%) was higher than those from the ‘poor’ (16.4%) and ‘middle’ (19.5%) households (*P = 0.016*). The rate also significantly increased with rising parity (*P = 0.023*). The rate among women who delivered in private health institutions (41.7%) was twice higher than their counterparts who delivered in public institutions (20.6%).

**Conclusion:**

The CS rate in Addis Ababa has exceeded beyond the level recommended by the WHO. Accordingly, It should be maintained within the optimum 5-15% range by introducing medical audit for labor management both in the private and public health institutions. Further, during prenatal care pregnant women should be fully informed about the risks of medically unjustified CS.

## Background

Caesarean Section (CS) is a surgical intervention designed to prevent or treat life threatening maternal or fetal complications. The capability to perform safe Caesarean delivery has been one of the major advancements in obstetrics in the 20^th^ century [1]. So far studies have availed empirical evidences on the importance of CS for the reduction of maternal and neonatal mortality. An ecological study found that in low-income countries, a negative and statistically significant linear correlation was observed between CS rate – the number of CS deliveries per 100 births – and neonatal mortality and between CS rate and maternal mortality [2]. Another ecological study also concluded that in countries with CS rates less than 15%, higher rates were associated to lower infant, neonatal, and maternal mortality rates [3]. On the contrary, high CS rates have not shown additional benefits, and even could cause negative maternal and child health consequences [4].

Though the appropriate range for the CS rate is debatable [5], the World Health Organization (WHO) considers 5-15% as the optimum range [6]. Nonetheless, in many middle and high income countries the rate is higher that the aforementioned limit and the opposite is being witnessed in low income countries. According to a 2007 estimate, CS rate was as high as 21.1% in developed and as low as 2% in least developed countries [7]. The WHO also estimated in 2008 that 3.2 million additional CS were needed in the developing world while 6.2 million needless Caesarean deliveries were performed elsewhere [8].

Globally in recent years the proportion of deliveries carried out by CS has risen considerably due to complex reasons including increase in women’s demand for the procedure [9,10]. The appropriateness and ethical aspects of on-demand CS has been hotly debated by obstetricians and women’s group for some years now. The debate has focused on the questions of risks and benefits of vaginal and CS delivery and woman’s autonomy to chose her mode of delivery [11]. One side discourages on-demand CS stressing that the procedure is not natural and it’s liable to surgical complications whereas the other emphasizes on the clients right for informed choice on the basis that risks, benefits, and costs are so balanced between Caesarean and vaginal delivery [11].

Caesarean delivery without proper medical indication can be associated with increased risk of adverse maternal and perinatal outcomes [12]. The WHO global survey witnessed that higher CS rates were associated with an increase in severe maternal morbidity and mortality, fetal mortality rates and higher numbers of babies admitted to intensive care for 7 days or longer [4]. A multi-country facility based survey concluded likewise [13]. A study based on the 1998 UK maternal mortality report showed that the case fatality rate for women with elective CS was 2.8-fold times higher than those who had a normal vaginal delivery [14].

In Ethiopia, large proportion of the population lacks access to essential obstetric care including CS [15]. The Ethiopia Demographic and Health Survey (EDHS) 2011 and 2005 reported exceptionally low national CS rates (1.5% and 1.0% respectively) [16,17]. Further, WHO estimated that in 2008 the total number of additional CS needed in Ethiopia in order to reach the minimum 5% rate was 278,370 and the figure was the third highest in the world [8]. However, the situation in Addis Ababa – the capital of Ethiopia – is the opposite. EDHS 2011 and 2005 reported 21.8% and 16.0% CS rates signifying the possibility of over-utilization of the service in the city [16,17]. Accordingly, the current study – based on EDHS data – was conducted in order to assess the trend and socio-demographic differentials of CS rate in the city.

## Methods

### Study design

The study was done based on the secondary data of EDHS. Differentials of CS were identified based on EDHS 2011 data; whereas, trend analysis was made using EDHS 2000, 2005 and 2011 data.

### Study setting

Addis Ababa is the largest and the capital city of Ethiopia. As of 2013 the city had 3.3 million inhabitants with male to female ratio of 0.91 [18]. Regarding medical service, currently the city has 41 hospitals, 28 health centers, 35 health posts and more than 500 clinics. According to EDHS 2011, the coverage of Antenatal Care (ANC), birth assistance by skilled provider and postnatal care in the city were 93.6%, 83.9% and 47.7%, respectively [16].

### Sample size

The trend analysis was made based on the available data of 1298 women (518, 380 and 400 from EDHS 2000, 2005 and 2011, respectively) who gave birth in the preceding 5 years of the surveys. On average for every data point (two consecutive years) 172 observations were available. Based on single proportion sample size calculation formula, at 95% confidence level, the available sample size was adequate to estimate 5-15% CS rate with 3-5% margin of error.

### Sampling method of EDHS

All the three DHS surveys used a multi-stage cluster sampling technique [16,17,19]. Initially Enumeration Areas (EA) — a cluster that conventionally encompasses 150–200 adjacent households — were selected as primary sampling units from the sampling frame developed based on the 1994 and 2007 censuses. Then in each of the selected EA a complete listing of households was carried out and ultimately households were drawn as secondary sampling units using systematic random sampling technique. In the three surveys, 51, 50, 54 EAs respectively were sampled from the city [16,17,19]. For this specific analysis, all data collected from women who gave birth in the preceding five years of the survey were included. At times when women had more than one birth in the reference period, the most recent one was considered.

### Data collection of EDHS

The EDHS data were collected by trained and experienced data collectors. The survey used a standard Measure DHS questionnaire adapted to the context of the country. The questionnaire was finalized in English and later translated into Amharic – the language widely spoken in the city. Prior to the fieldwork, the tool was pretested and necessary modifications were made [16,17,19].

### Data analysis

The data were analyzed using SPSS 20.0 for windows. CS rate was compared across various categories of socio-economic variables using chi-square analysis. Pearson’s chi-square and chi-square for trend analyses were used for nominal and ordinal variables, respectively.

The DHS surveys collected information about mode of delivery of the recent birth that happened in the preceding 5 years period. In this analysis, based on the specific year of delivery, the rate was calculated for each year from 1995 to 2010. As the year 2005 had not been included in any of the surveys, its rate was interpolated based on 2004 and 2006 figures. In order to improve the adequacy of the sample size for estimating the rate for every data point, CS rates were recalculated by merging the observation of every two consecutive years into one. On average, for every data point 172 observations were available.

The trend of CS rates was assessed using simple linear regression analysis. Prior to the analysis the absence of auto-correlation was checked using Durbin-Watson test. Other assumptions of the analysis (linearity, normally distributed and homoscedastic error terms) were also satisfied. The statistical significance of the trend was evaluated using t-test.

Wealth tertiles (poor, middle and rich) were generated using principal component analysis. The analysis was made based on 16 variables related to ownership of selected household assets and materials used for housing construction.

### Ethical consideration

The datasets were accessed after taking permission from Measure DHS. The primary data were collected in line with national and international ethical guidelines. Ethical clearance was provided by the Institutional Review Boards of Ethiopian Health and Nutrition Research Institute, Ministry of Science and Technology of Ethiopia, ICF International, and the CDC [16].

## Results

### Socio-demographic characteristics

In DHS 2011, information about Caesarean delivery was collected from 400 women who gave their recent birth in the preceding 5 years of the survey. The mean age (±SD) of the respondents was 28.2 (±5.3) years and 62.5% were between 25–34 years of age. The median parity was 2 and it ranged from 1 to 8. Nearly four-fifth (78.0%) had some form of formal education and more than half (57.5%) were unemployed at the time of the survey (Table 1).

**Table 1:** Socio-demographic characteristics of women who gave their recent birth in the preceding 5 years of the DHS 2011 survey, Addis Ababa, Ethiopia, 2011

**Socio-demographic characteristics (n = 400)**	**Frequency**	**Percent**
Age (years)		
18-24	92	23.0
25-34	250	62.5
35-44	58	14.5
Parity		
1	162	40.5
1-4	203	50.8
5 or more	35	8.8
Education status		
Illiterate	88	22.0
Primary education	171	42.8
Secondary education	93	23.3
Higher education	48	12.0
Employment status		
Employed	170	42.5
Unemployed	230	57.5
Marital status		
Married/living together	349	87.3
Divorced/separated	32	8.0
Single	15	3.8
Widowed	4	1.0

### Trends of Caesarean section rate

According to the DHS survey reports, the CS rate over the preceding 5 years of the surveys had increased considerably from the level of 8.2% (95% Confidence Interval (CI): 6.0-10.9) in 2000 to 15.3% (95% CI: 11.8-19.3) and 21.5% (95% CI: 17.5-25.9) in 2005 and 2011, respectively. Though the CIs for the 2005 and 2011 surveys overlapped, based on chi-square for trend analysis, the overall increment was statistically significant *(X*^*2*^ *= 32.617, P < 0.001)*. As compared to the figure reported in 2000, the CS rate in 2011 had increased by 2.6 folds.

**Figure 1:**
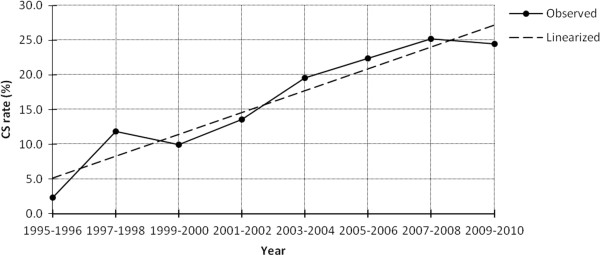
Trends of Caesarean section rate in Addis Ababa, Ethiopia, 1995–2010.

Year specific figures illustrated that the CS rate had increased from 2.3% in 1995–1996 to 24.4% in 2009–2010. Based on the linear regression analysis, the rise was statistically significant (*t = 8.066, P < 0.001*). The regression analysis also found that on average, over the 16 years period (1995–2010), the figure increased at an annual rate of 1.6% (Figure 1). From 2003 onwards, the rates persisted above the level of 15%.

### Differentials of Caesarean section rate

The CS rate increases with rise in maternal education and household wealth index. The rates among women with secondary (32.3%) or higher (33.3%) levels of education were nearly two times higher than the corresponding figures in illiterates (14.8%) and women with primary education (15.8%) (*P < 0.001*). The CS rate among women from the ‘rich’ households (28.6%) was significantly higher than those from the ‘poor’ (16.4%) and ‘middle’ (19.5%) households (*P = 0.016*).

Of all 400 respondents, 319 gave their recent birth either in private for-profit (clinics or hospitals) or public (health centers or hospitals) health facilities. The CS rate among women who delivered in private health facilities (41.7%) was twice increased as compared to women who gave birth in the public health facilities (20.6%) (*P < 0.001*) (Table 2).

**Table 2:** Caesarean section rate across various characteristics of the respondents, Addis Ababa, Ethiopia, 2011

**Variables**	**Caesarean section**		**X**^**2**^**and *p *** **value**
**(n = 400)**	**Frequency**	**Rate**	
Age (years)			
18-24 (n = 92)	20	21.7	*X*^*2*^ = 0.214, *P* = 0.643
25-34 (n = 250)	51	20.4	
35-44 (n = 58)	15	25.9	
Parity			
Primiparous (n = 162)	43	26.5	*X*^*2*^ = 5.159, *P* = 0.023*
Multiparous (n = 203)	39	19.2	
Grand-multiparous (n = 35)	4	11.4	
Educational status			
Illiterates (n = 88)	13	14.8	*X*^*2*^ = 12.662, *P* < 0.001*
Primary (n = 171)	27	15.8	
Secondary (n = 93)	30	32.3	
Higher education (n = 48)	16	33.3	
Employment status			
Employed (n = 170)	36	21.2	*X*^*2*^ = 0.180, *P* = 0.892
Unemployed (n = 230)	50	21.7	
Household wealth index			
Poor (n = 134)	22	16.4	*X*^*2*^ = 5.820, *P* = 0.016*
Middle (n = 133)	26	19.5	
Rich (n = 133)	36	28.6	
Place of birth			
Public health institutions (n = 247)	51	20.6	*X*^*2*^ = 36.798, *P* < 0.001*
Private for-profit health institutions (n = 72)	30	41.7	

*Significant at p value of 0.05.

The CS rate declines with increasing parity (*P = 0.023*). The highest rate of 26.5% was reported among primiparous women. The corresponding figures for multiparous and grand-multiparous were 19.2% and 11.4%, respectively. On the other hand, the CS rate did not show significant variation across the categories of maternal age and employment status (*P > 0.05*).

## Discussion

According to the WHO, the CS rate in any population should lie within the range of 5-15% and there is no justification in any specific geographic region to have more than 10-15% CS births [6,20]. However, the current study has demonstrated that in Addis Ababa the rate had increased significantly between 1995 and 2010 and it persisted above 15% since 2003.

Ethiopia, alike most of the developing countries, is characterized by inadequate access to CS service. The recent DHS 2011 indicated that only 1.5% of all births in the country were Caesarean deliveries [16]. Another large-scale institution based study estimated a national rate of 0.6% that ranged from 0.2 to 9% across the sub-national regions [21]. The earlier two DHS surveys also came up with extremely low figures [17,19]. Compared to the aforementioned studies, the relatively high CS rate found in Addis Ababa clearly indicate that national level figures can mask within-country variation and can at times mislead public health interventions. Consequently, collection of sub-national data or disaggregation of national figures is of enormous significance.

Based on the linear regression analysis, between 1995 and 2010, the prevalence of CS had been increasing with an annual rate of 1.6%. Elsewhere, few studies have also documented such rapid rate of increment. A hospital based study in Nairobi, Kenya reported a rise from 20.4% in 1996 to 38.1% in 2004 with an equivalent rate of 2% per year [22]. In Ribeirao Preto, Brazil the CS prevalence increased from 30.3% in 1978 to 50.8% in 1994 with the approximate annual rate of 1.2% [23]. Likewise, in Hong Kong from 1987 to 1999 the prevalence rose steadily from 16.6 to 27.4 with the rate of 0.8% per year [24].

Although the CS rates were elevated in all socioeconomic groups, the procedure was more frequent among socio-economically privileged women. According to a study in China, educated women were 3–4 times more likely to have CS as compared to illiterates; further, women from the upper income quartile had 3 fold increased probability of CS than those from the lowest quartile [25]. Studies in Brazil and Italy also concluded the same [26,27]. At times women may consider CS as less painful, convenient and safer option than vaginal delivery [10]; hence, the socio-economically empowered women who have limited financial barriers may over-utilize the service.

The results show that the CS rate among women who delivered in the private for-profit health institutions was considerably high (41.7%) and it was also two times higher than the rate in the public institutions. Likewise, a national study conducted in 2008 in Ethiopia found 3 times higher CS rate in the private sector [21]. The finding can be due to various reasons. Firstly, private institutions may conduct CS without clear-cut medical indications or on maternal request in order to satisfy their clients’ demand. Secondly, as the service provided by the private sector is commonly perceived to be of better quality, mothers with complications that genuinely need CS may often prefer them.

Based on the estimated crude birth rate and population size of Addis Ababa [18], in 2010 approximately 44,300 births had taken place in the city. Considering the 24.4% CS rate computed for the specific year, approximately 10,721 Caesarean births had happened in 2010. Consequently, taking 15% as the maximum optimum CS rate, in 2010 alone roughly 4,076 unnecessary CS were performed in the city.

A study conducted by the WHO in 2010 [8], estimated the global cost of unnecessary and extra needed CS for the year 2008. The unit marginal cost of the procedure was predicted by considering costs associated with the medical supplies, post-operative hospital stay, human resources time and management of potential medical complications. Ultimately country specific unit values were determined and used to estimate the global cost. In the study, the unit cost calculated for Ethiopia was 132.7 US dollars per procedure. Considering this cost as a valid estimate, the 4,076 unnecessary procedures conducted in Addis Ababa in 2010 might have incurred around 540,885 US Dollars (10,276,815 Ethiopian Birr).

In general unlike many previous undertakings, this study assessed the differentials of CS rate based on community based data and assessed the trend over a reasonable period of time. Conversely, some limitations need to be considered while interpreting the findings of the study. Differentials of CS rate were identified based on bivariate analysis hence confounding cannot be excluded. The available sample size for each data point was adequate to estimate the CS rate with 3-5% margin of error; however, smaller margin of error would have been more optimal for the study. Further, at times when mothers had more than two births in the reference period, only the recent one was considered for the analysis and this could have introduced selection bias in the study.

## Conclusion

The CS rate in Addis Ababa has increased considerably from 2.3% in 1995–1996 to 24.4% in 2009–2010. Since 2003 the rate persisted beyond the upper optimum level of 15%. The CS rate significantly increased with a rise in maternal education and household wealth index; whereas it decreased with increasing parity. The rate among women who delivered in private health facilities was twice higher than those who gave birth in the public health facilities.

The CS rate should be maintained within the optimum range by introducing medical audit of labor management both in private and public health facilities. Furthermore, expectant women should be fully informed about the risks associated with medically unjustified Caesarean section.

## Competing interests

The author declares that he has no competing interests.

## Authors’ information

SG: Assistant professor of public health, Hawassa University, Ethiopia.
